# Beyond the Surface: A Methodological Exploration of Enzyme Impact along the Cellulose Fiber Cross-Section

**DOI:** 10.1021/acs.biomac.4c00152

**Published:** 2024-04-18

**Authors:** Irina Sulaeva, Fredrik Gjerstad Sto̷pamo, Ivan Melikhov, David Budischowsky, Jenni L. Rahikainen, Anna Borisova, Kaisa Marjamaa, Kristiina Kruus, Vincent G. H. Eijsink, Anikó Várnai, Antje Potthast

**Affiliations:** † Core Facility Analysis of Lignocellulosics (ALICE), University of Natural Resources and Life Sciences, Vienna (BOKU), Konrad Lorenz-Strasse 24, A-3430 Tulln an der Donau, Austria; ‡ Faculty of Chemistry, Biotechnology and Food Science, NMBU − Norwegian University of Life Sciences, 1432 Ås, Norway; § Institute of Chemistry of Renewable Resources, Department of Chemistry, University of Natural Resources and Life Sciences, Vienna (BOKU), Konrad Lorenz-Strasse 24, A-3430 Tulln an der Donau, Austria; ∥ Solutions for Natural Resources and Environment, VTT Technical Research Centre of Finland Ltd., Tietotie 2, FI-02044 Espoo, Finland; ⊥ School of Chemical Engineering, Aalto University, P.O. Box 16100, 00076 Espoo, Finland

## Abstract

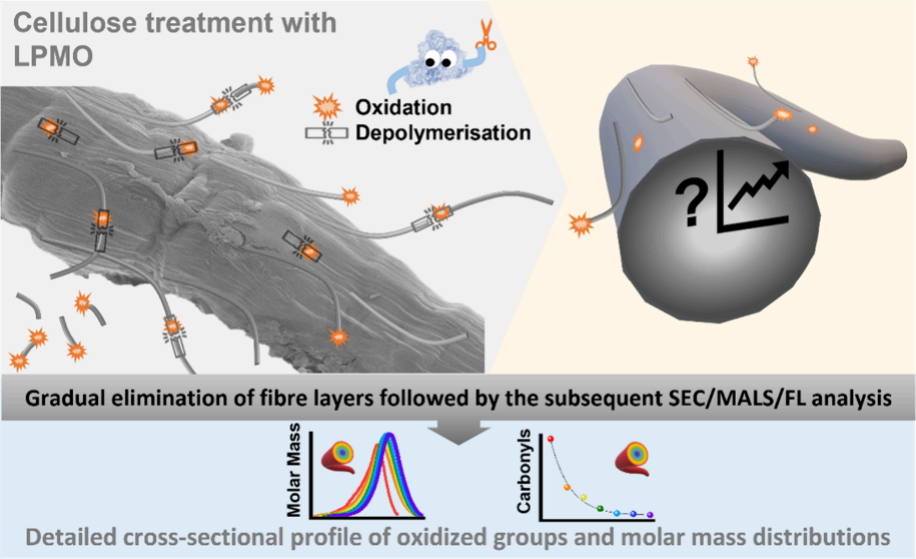

Despite the wide range of analytical tools available for the characterization of cellulose, the in-depth characterization of inhomogeneous, layered cellulose fiber structures remains a challenge. When treating fibers or spinning man-made fibers, the question always arises as to whether the changes in the fiber structure affect only the surface or the entire fiber. Here, we developed an analysis tool based on the sequential limited dissolution of cellulose fiber layers. The method can reveal potential differences in fiber properties along the cross-sectional profile of natural or man-made cellulose fibers. In this analytical approach, carbonyl groups are labeled with a carbonyl selective fluorescence label (CCOA), after which thin fiber layers are sequentially dissolved with the solvent system DMAc/LiCl (9% w/v) and analyzed with size exclusion chromatography coupled with light scattering and fluorescence detection. The analysis of these fractions allowed for the recording of the changes in the chemical structure across the layers, resulting in a detailed cross-sectional profile of the different functionalities and molecular weight distributions. The method was optimized and tested in practice with LPMO (lytic polysaccharide monooxygenase)-treated cotton fibers, where it revealed the depth of fiber modification by the enzyme.

## Introduction

In the transition to a more sustainable future, which is now a priority goal for countries around the world, cellulose is seen as a key polymer for green, biobased development. As part of biomass, cellulose is a renewable source of biobased polymers and helps to reduce the consumption of fossil resources.^1^ In its polymeric form, cellulose is today mainly used in pulp, paper, and textile production. Efforts are being made worldwide to further develop biobased processes that will enable cellulose to be used on a much larger scale.

Despite its abundance, some challenges restrict the utilization of cellulose as an industrial feedstock. Its complex hierarchical structure often renders it resistant to mild chemical and mechanical treatments. To reduce the consumption of harsh chemicals and ensure the environmental compatibility of cellulose processing, an enzymatic treatment is frequently implemented. Efficient enzymatic cocktails–mixtures of cellobiohydrolases and endoglucanases in combination with a variety of hemicellulases and auxiliary enzymes–are already extensively utilized in biorefinery processes for the complete hydrolysis of lignocellulosic biomass for production of biofuels.^2^ One of the more recent developments in this field concerns the discovery of lytic polysaccharide monooxygenases (EC 1.14.99.53–56, LPMOs), which can break internal glycosidic linkages even within the most recalcitrant crystalline parts of cellulose, introducing oxidized sites. These enzymes are considered key enzymes that increase the efficiency of cellulose-hydrolyzing enzymes in biomass degradation,^3^ while also offering possibilities for cellulose modification.^4^ LPMO-catalyzed oxidation occurs at the C1 and/or C4 carbon of the anhydro glucose units in a cellulose substrate and requires the presence of an external electron donor and an oxygen species. LPMOs show broad catalytic versatility, acting on multiple soluble and insoluble substrates.^5^

Despite the obvious and unique potential of LPMO enzymes in cellulose modification, they have been underexplored for controlled fiber treatment.^6^ In contrast to total saccharification, the use of enzymes in fiber engineering requires targeted surface-only modifications as unrestricted treatment by an enzyme is known to weaken the fibers due to cellulose depolymerization. As the mechanism and impact of LPMO action on the cellulose surface are still not well understood, it is challenging to predict the effects of LPMO treatment, for example, whether it results in surface-only modification of cellulosic fibers without deep penetration to the fiber core or in extensive cellulose degradation with effects on deeper layers.

LPMOs are known to feature a flat, sometimes slightly grooved substrate binding surface with an exposed copper-containing active site.^7,8^ However, the exact amino acid residues on the binding surface responsible for substrate recognition and oxidative regioselectivity of LPMOs are still poorly understood.^9,10^ In addition to a catalytic module, some LPMOs possess a carbohydrate-binding module (CBM type 1) connected via a flexible linker to the C-terminus,^11^ which influences their enzymatic activity due to the effects on substrate binding.^11,12^ The effects of CBMs on LPMO activity are complex, as the affinity for the substrate helps to prevent autocatalytic inactivation of the enzyme under turnover conditions,^13^ as discussed below. Real-time imaging of the molecular interactions of a CBM-containing LPMO (*Tr*AA9A from *Trichoderma reesei*) with bacterial microcrystalline cellulose performed using high-speed atomic force microscopy has shown that *Tr*AA9A molecules exhibit a “stop-and-go” behavior in all three dimensions, including movement along one cellulose microfibril, across it, or from one microfibril to another. Moreover, enzyme molecules have been shown to penetrate inside the microfibril structure.^14^

For the successful optimization of the fiber processing parameters, it is critical to gain a better in-depth understanding of the underlying mechanisms, including enzyme–fiber interaction and enzymatic fiber penetration. In this respect, it would be helpful to have access to methods that allow monitoring cellulose properties along the cross-section of the fiber or for successive thin fiber layers, including properties such as molar mass distribution and occurrence of functional groups like carbonyls or carboxyls. The concept of performing cross-sectional fiber characterization is not novel. Subsequent fiber layer analysis has been explored previously to gain insights on the cross-sectional variation of specific parameters, such as chemical composition, including the distribution of lignin^15−17^ and carbohydrates,^16,18−20^ as well as changes in molar mass.^20,21^ Various techniques can be applied to isolate individual fiber layers, including mechanical peeling,^15−17,22^ enzymatic peeling,^18,19^ and chemical peeling.^20^ However, most of the developed, mechanical, and enzymatic peeling approaches are not suitable for analyzing LPMO–fiber interactions because they rely on cellulose degradation during peeling and thus fail to provide valuable information about the degree of cellulose chain cleavages induced by LPMO. Chemical peeling involves derivatization (acetylation) of carbohydrates at the fiber surface and their subsequent extraction by organic solvents. This technique has never become widely applied because of challenges, such as poor reproducibility and time inefficiency. Moreover, derivatization reactions during chemical peeling introduce changes to the molar mass, restricting further analysis of original molar mass parameters for isolated fractions. Alternatively, gradual elimination of cellulose fiber layers was performed upon short-time dissolution in the most commonly used solvent system for cellulose, *N,N*-dimethylacetamide (DMAc) with LiCl (9% w/v).^21^ Among the available techniques for stepwise elimination of fiber layers, dissolution in DMAc/LiCl is the most suitable method for the intended purpose, as it preserves cellulose molar mass in the collected fractions. Moreover, fractions in DMAc can be directly analyzed by size-exclusion chromatography (SEC) to observe radial changes of molar masses introduced by LPMO in cellulose fibers. For a more comprehensive characterization, changes in carbonyl group content for each fiber layer can be recorded using a recently described protocol.^23^

This study evaluates a method for simultaneous analysis of changes in molar mass and carbonyl groups in cellulose fibers across all fiber layers from the surface to the core. The complete protocol includes labeling of cellulose samples with the carbonyl-selective fluorescent marker carbazole-9-carboxylic acid [2-(2-aminooxyethoxy)ethoxy]amide (CCOA), gradual peeling of fiber layers using DMAc/LiCl, followed by SEC analysis combining multi angle light scattering (MALS), fluorescence (FL), and refractive index (RI) detection. This new method was used to analyze LPMO-treated fibers in order to enhance our understanding of how LPMOs affect fiber properties. To get a better insight into the role of CBMs, two LPMOs belonging to the fungal AA9 family^24^ C1/C4-oxidizing *Tr*AA9A from *Trichoderma reesei* and C4-oxidizing *Nc*AA9C from *Neurospora crassa*, were studied in their native form and in a truncated form lacking the CBM. Thus, this study sheds light on how LPMOs and their CBM modify cellulose fibers. The cross-sectional analysis developed here represents a general-purpose tool that can be further exploited for the analysis of different layered cellulose structures.

## Materials and Methods

### Enzyme Production

*Tr*AA9A from *T. reesei* (UniProt ID, G0R6T8)^24^ was produced and purified as described in Kont et al.^25^ The truncated form of *Tr*AA9A, i.e., lacking the native CBM1, was produced by papain cleavage of the linker as follows: 53 mg of purified *Tr*AA9A (3.5 mg/mL) was treated with papain (0.3 mg/mL) in 0.1 M sodium acetate buffer, pH 5.0, at 37 °C for 23.5 h. The digested protein sample was changed to 10 mM sodium phosphate buffer, pH 7.0, with a PD10 desalting column (Cytiva, Marlborough, Massachusetts). The *Tr*AA9A catalytic domain was purified with anion exchange chromatography using a 5 mL DEAE Sepharose column (Cytiva, Marlborough, MA, USA) and a kta Protein purification system (Cytiva, Marlborough, MA, USA). The protein was eluted by applying a 0–150 mM sodium chloride gradient in 10 mM sodium phosphate buffer, pH 7.0, over 15 column volumes. The fractions were analyzed with SDS-PAGE (BioRad Criterion Stain Free Gel Imaging System; Bio-Rad Laboratories, Inc., Hercules, CA, USA), and fractions containing pure protein with the correct molar mass were pooled and concentrated with VivaSpin Turbo ultrafiltration tubes (cut off 5 kDa) (Sartorius, Goettingen, Germany) and changed to 25 mM sodium acetate buffer, pH 5.0, using a PD10 column. The purified enzymes were stored frozen (−20 °C). Prior to use, the purified catalytic domain of *Tr*AA9A (*Tr*AA9A-N) was saturated with copper by changing the buffer to 20 mM Tris–HCl, pH 8.0, with a PD10 column, followed by incubation with a 3-fold molar concentration of CuSO_4_ for 30 min at room temperature. After this, the excess copper sulfate was removed and the enzyme was changed back to 25 mM sodium acetate buffer, pH 5.0, with a PD10 desalting column. Protein concentrations were analyzed by measuring absorbance at 280 nm and converting these to molar concentrations using the theoretical molar extinction coefficient 54360 M^–1^·cm^–1^ for *Tr*AA9A and 48150 M^–1^·cm^–1^ for *Tr*AA9A-N.

*Nc*AA9C from *N. crassa* (UniProt ID, Q7SHI8) was produced and purified as reported by Kittl et al.^26^ The truncated, CBM-free version of *Nc*AA9C (*Nc*AA9C–N) was cloned, produced and purified as reported by Borisova et al.^27^

The cellobiohydrolase *Tr*Cel7A from *Trichoderma reesei* (UniProt ID, G0RVK1) was produced and purified as reported by Ståhlberg et al.^28^ The cellobiose dehydrogenase MtCDH from *Myriococcum thermophilum* (UniProt ID, A9XK88) was produced and purified as reported by Zámocký et al.^29^

### Cellulose Treatment with LPMO

Whatman No. 1 filter paper sheets purchased from GE Healthcare (production site China) were cold disintegrated and washed to obtain the sodium form as described by Marjamaa et al.^30^ For treatment with *Tr*AA9A and *Tr*AA9A-N, the wet cellulosic fibers were treated with 1.6 μM enzyme at 2.5% (w/v; dry matter) fiber concentration (0.064 μmol of enzyme/g of dry fiber) in 50 mM sodium phosphate buffer, pH 7.0, containing 1 mM gallic acid (GA) as reductant. The reactions were carried out in 100 mL glass bottles covered with pierced aluminum foil at 45 °C for 6 h with mixing at 170 rpm using an Infors HT Ecotron incubator shaker (Infors AG, Bottmingen, Switzerland). Each reaction contained 0.5 g (dry matter) fibers in a 20 mL liquid volume. After the reaction, the bottles were placed in an ice bath, and the liquid was collected by filtration through a 60 μm mesh cloth. The filtrate was poured onto the fibers on the mesh cloth, and the filtration was repeated. The filtrate was then collected and stored at −20 °C. The LPMO-treated fibers were further washed with 100 mL of Milli-Q water by filtration. The washed fiber samples were then stored at 4 °C. Control reactions were set up, with one lacking the enzyme and another lacking GA.

For treatment with *Nc*AA9C and *Nc*AA9C–N, the wet cellulosic fibers were treated with 0.5 μM enzyme at 1% (w/v; dry matter) fiber concentration (0.05 μmol of enzyme/g of dry fiber) in 50 mM Bis-Tris/HCl buffer, pH 6.5, containing 1 mM GA as reductant. The reactions were carried out in 50 mL falcon tubes at 30 °C for 24 h with mixing at 250 rpm horizontal shaking using an Infors HT Multitron Standard incubator shaker (Infors AG). Each reaction contained 50 mg (dry matter) fibers in a 5 mL liquid volume. The reactions were terminated by incubating at 99 °C for 5 min, and the solids were separated from the liquid fraction by centrifugation at 5000*g* and 4 °C for 20 min and removing the supernatant by pipetting. Subsequently, 200 μL of the supernatant was filtered through a 0.2 μm PES membrane using a 96-well filter plate and a vacuum manifold (Merck Millipore; Burlington, MA, USA) for analysis of the soluble LPMO products. Control reactions were set up, with one lacking the enzyme and another lacking GA.

To remove residual proteins after LPMO treatment, the recovered fiber fractions (ca. 50 mg dry material each) were immediately resuspended in 5 mL of 1% (w/v) sodium dodecyl sulfate (SDS) solution and boiled for 5 min, then washed three times with 5 mL 75% (w/v) EtOH and once with 5 mL Milli-Q water, and then resuspended with 15 mL 75% (v/w) EtOH prior to fiber analysis, with centrifuging (at 5000*g* and 4 °C for 20 min) and then removing the supernatant by pipetting after each round.

### Analysis of Soluble Products

Soluble LPMO products were analyzed using high-performance anion exchange chromatography with pulsed amperometric detection (HPAEC-PAD) using a Dionex ICS-5000 system (Thermo Scientific, Sunnyvale, CA, USA) equipped with a CarboPac PA200 analytical (3 × 250 mm) and guard (3 × 50 mm) column, using a 26 min gradient protocol reported earlier.^31^ Before analysis, the samples were treated with 1 μM TrCel7A in 20 mM Na-acetate buffer, pH 5.5, overnight at 37 °C, to convert oligomeric oxidized products to a mixture of Glc4gemGlc, Glc4gem(Glc)2, GlcGlc1A, (Glc)2Glc1A, and (Glc)3Glc1A. C1-oxidized standards (GlcGlc1A, (Glc)2Glc1A, and (Glc)3Glc1A) were produced by treating cellobiose, cellotriose, or cellotetraose (1.25 mM) with cellobiose dehydrogenase MtCDH (2 μM) in 50 mM sodium acetate buffer, pH 5.0, at 40 °C for 24 h. We synthesized C4-oxidized standards, Glc4gem(Glc)2 and Glc4gemGlc, by treating cello-1,4-β-D-pentaose (Megazyme International, Ireland) with C4-oxidizing *Nc*AA9C, as described previously.^32^

### Cellulose Carbonyl Group Labeling with CCOA

Cellulose labeling with CCOA was performed according to a procedure described before.^33^ In short, 100 mg of pulp was suspended in 10 mL of a 20 mM zinc acetate buffer (pH 4.0) containing 12.5 mg CCOA and incubated for 7 days at 40 °C. The labeled pulp was isolated by filtration, washed with EtOH and DMAc, and subjected to dissolution using the methods described below.

### Stepwise Dissolution

#### Approach I

**Figure 1:**
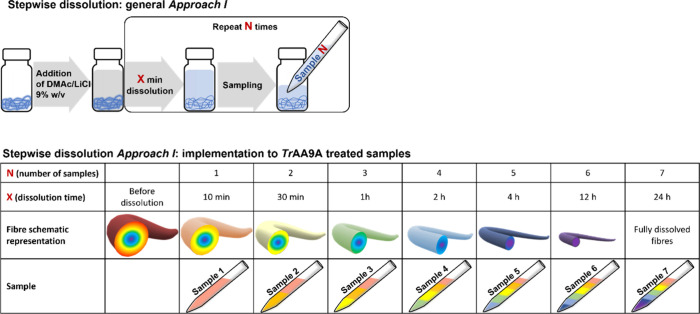
Schematic representation of stepwise dissolution **Approach I** and the details of its implementation to the analysis of *Tr*AA9A-treated samples.

A sample (approximately 50 mg corresponding to air-dry weight) was placed into a 15 mL vial, and DMAc/LiCl (9% w/v, 7 mL) was added to the sample. The vial was vortexed for 5 min and kept in a shaker. Samples (1 mL each) were taken from the vial after defined time intervals (10 min, 30 min, 1, 2, 4, 12, and 24 h), as shown in Figure 1. Each sample was diluted with pure DMAc (1:1, v/v) and filtered through a 0.45 μm syringe filter prior to SEC analysis. With this approach, the subsequent samples contain an increasing fraction, i.e., an increasingly thicker part of the outer layer) of the fibers.

#### Approach II

**Figure 2:**
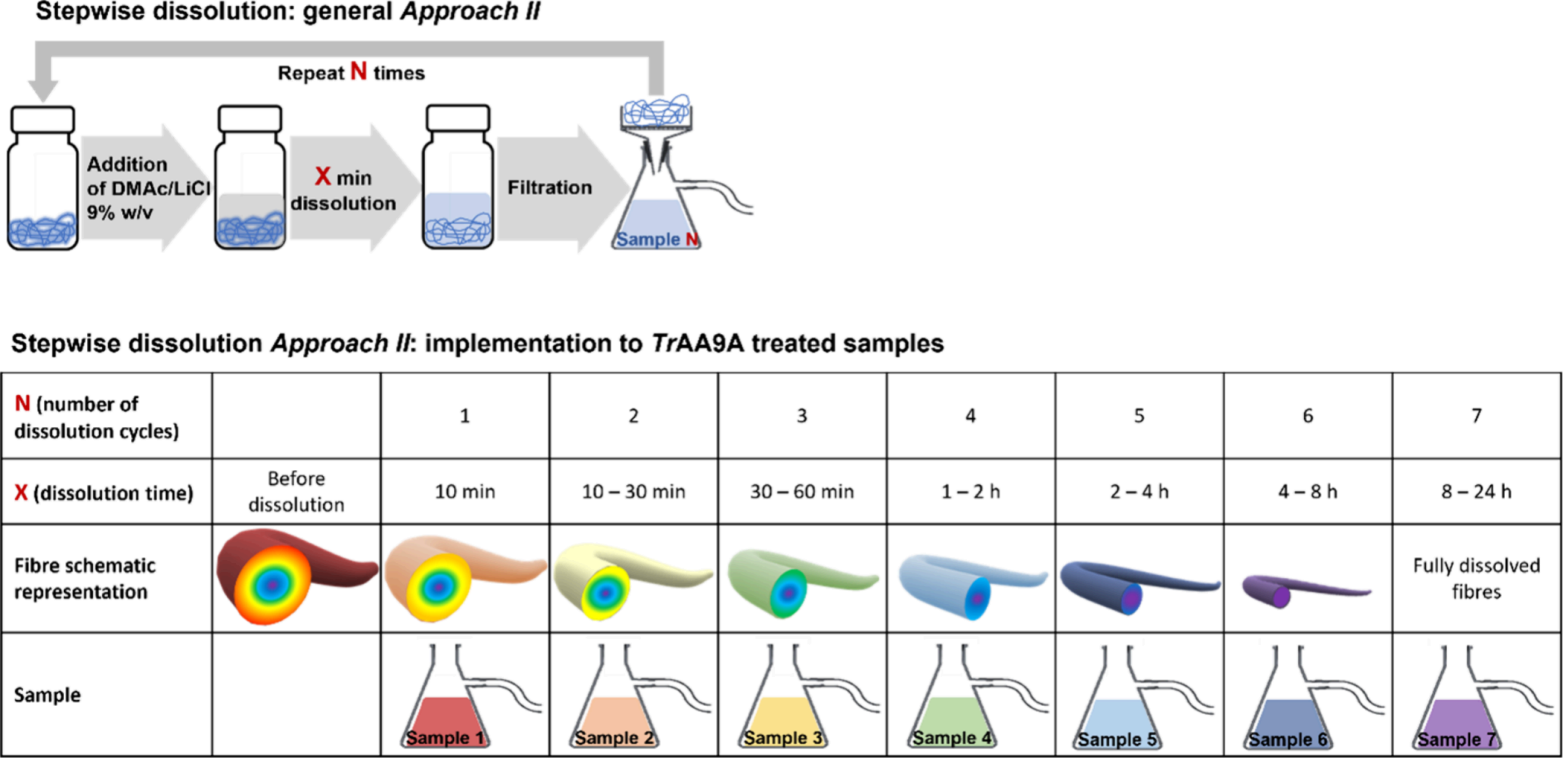
Schematic representation of stepwise dissolution **Approach II** and the details of its implementation to the analysis of *Tr*AA9A-treated samples.

A sample (approximately 50 mg corresponding to air-dry weight) was placed into a 15 mL vial, and DMAc/LiCl (9% w/v, 1 mL) was added. The sample was first vortexed for 5 min and then kept in a shaker. After certain time intervals, the sample was diluted with 1 mL of pure DMAc to stop the dissolution reaction. The diluted sample was filtered on a Büchner funnel connected to a 25 mL flask. The permeate containing the dissolved outer layer of the cellulose fibrils was collected, filtered through a syringe filter, and subjected to SEC analysis. The retentate-containing fibers without the now removed outer layer were flushed with pure DMAc, carefully collected from the filter, and placed in a 15 mL vial for further dissolution. After the next addition of 1 mL of DMAc/LiCl (9% w/v), the sample was stirred for a defined period of time, after which the filtering procedure was repeated. This dissolution/filtration cycle was repeated a few times, as shown in Figure 2, to collect samples with total dissolution times of 10 min, 30 min, 1, 4, 8, and 24 h. In contrast to the first stepwise dissolution approach, the samples collected using this method contain fractions belonging to a specific time interval and, thus, a specific depth interval of the fiber.

### SEC Analysis

SEC system components: G1312B HPLC pump (Agilent Technologies, Waldbronn, Germany); G1367B autosampler (Agilent Technologies, Waldbronn, Germany); TSP FL2000 fluorescence detector (excitation: 290 nm, emission: 340 nm); Wyatt Dawn DSP MALS detector with a laser diode (λ = 488 nm) (Wyatt Technology, Santa Barbara, US); Shodex RI-71 refraction index (RI) detector (Showa Denko Europe GmbH, Munich, Germany). DMAc/LiCl (0.9%, w/v), filtered through a 0.02 μm filter, was used as the eluent. The following parameters were used in SEC: Flow: 1.00 mL/min; columns: four Agilent MIXED-ALS columns, 20 μm, 7.5 mm × 300 mm (Agilent Technologies, Waldbronn, Germany); Injection volume: 100 μL; Run time: 45 min. Data were evaluated with the Astra 4.73, Grams 7, Access, and OriginPro 2020 software packages. The experimental uncertainties were validated from multiple analyses (ten different batches) of untreated reference material and calculation of relative standard deviations (RSD) for *M*_*n*_, *M*_*w*_, and *M*_*z*_; The detailed results of RSD analysis for these parameters are given in Table S1 and Figure S1. The RSD value for total carbonyl numbers (2.88%) was taken from the original paper of Röhrling et al.^33^

### Scanning Electron Microscopy (SEM)

A benchtop scanning electron microscope (JEOL JCM-6000, JEOL Ltd., Tokyo, Japan) was used to investigate the fiber morphology. Fibers were fixed to the sample holder using conductive carbon stickers (Agar Scientific Ltd., Essex, UK). The samples were gold-coated in an argon atmosphere using a fine coater (JEOL JFC-1200, JEOL Ltd.). SEM micrographs were collected at various magnifications from numerous sites in each sample.

## Results and Discussion

### Dissolution of Cellulose Fibers

**Figure 3:**
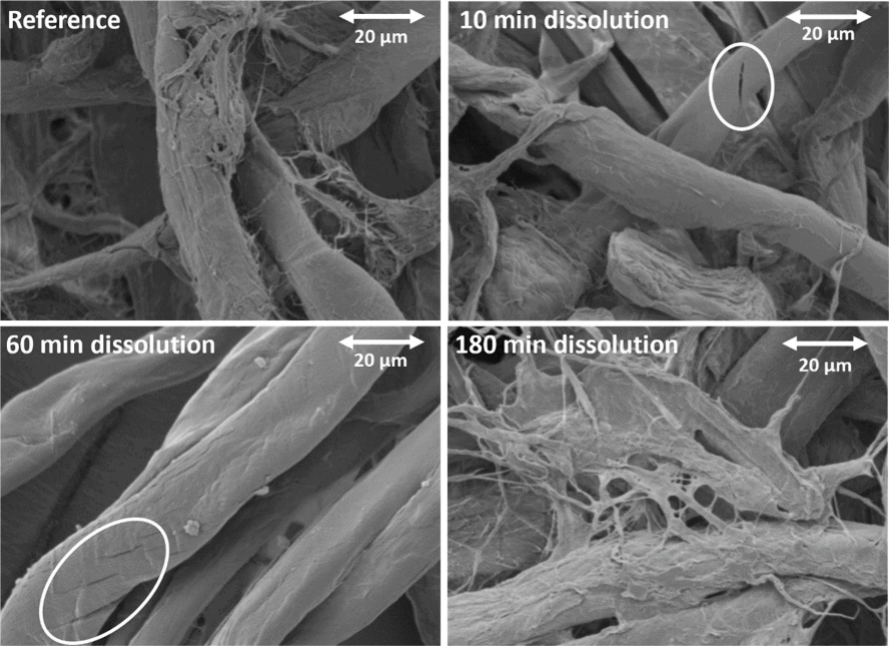
SEM pictures of Whatman No. 1 cotton fibers during dissolution in DMAc/LiCl (9% w/v). The pictures, taken at different time points after starting the dissolution and show the gradual elimination of fiber layers from the surface. Cracks in the fiber primary wall and at the first layer of the secondary wall (P/S1 layer) are visible within the white frames.

The structural integrity of cellulose, caused by a particularly strong hydrogen bond network, is one of the reasons for limited cellulose solubility in the most common solvents. In SEC analysis of cellulose, which requires the fully dissolved state, the DMAc/LiCl solvent system is one of the most used, as it dissolves celluloses of different molar masses without the need for derivatization, does not cause degradation, and results in a stable solution under ambient conditions. Moreover, the dissolution mechanism of cellulose fibers in DMAc/LiCl has been thoroughly studied,^34,35^ and changes in fiber morphology upon dissolution can be predicted depending on the pulp type.^21,36,37^ For example, it has been shown that in most pulps from annual plants, the readily accessible outer regions (i.e., fiber surface), which contain both low molar mass hemicellulose and cellulose, are dissolved first, followed by the slower dissolution of more structured cellulose layers.^21^ Cotton fiber from Whatman No. 1 filter paper, which has been used in the present study, does not contain considerable amounts of hemicelluloses.^38^ Thus, their dissolution is primarily influenced by the cellulose fiber morphology. The gradual dissolution of cotton fibers in DMAc/LiCl was visualized using SEM (Figure 3).

The SEM images show that the dissolution starts by exfoliation of readily accessible fibrils that disappear from the fiber surface already after 10 min of dissolution. The elimination of accessible fibrils from the outer layers continues with time. As dissolution progresses, fine cracks appear on the fiber primary wall (P-layer) and further at the first layer of the secondary wall (S1-layer), facilitating penetration of the solvent. Diffusion of solvent through the cracks in the P/S1 layer opens the fiber and makes the next part of the fibril accessible for dissolution, as can be seen after 180 min of continued dissolution. This correlates with previous findings where molar mass distribution curves recorded during the progressive dissolution of Whatman fibers have shown gradual solubilization of outer layers caused by progressive penetration of the solvent to the fiber core.^21^ In this work, this layer-by-layer dissolution mechanism is enhanced further by isolating the entire dissolved fraction after each dissolution step, thereby generating solubilized fractions corresponding to specific layers of the cellulose fibril. Characterization of such fractions, covering the cellulose fibril from surface to core, could potentially provide insight into how external factors, for example, the action of an enzyme such as LPMO, affect the cellulose fibril.

### Comparison of Two Gradual Dissolution Approaches Using LPMO-Treated Cellulose

The initial time intervals (10 min to 4 h) applied for the dissolution of cellulose fibers were generally designed to be short enough to avoid complete fiber dissolution. In the first gradual dissolution approach (**Approach I**), the fractions collected after short dissolution times contained exclusively the outer layers of cellulosic fibers. The consecutive fractions will contain increasing amounts of material derived from layers that are further away from the fiber surface and closer to the fiber core, and comparative analysis of consecutive fractions will thus show how fiber properties change when moving from surface to core (Figure 1). To assess this method, cellulose fibers were treated with full-length *Tr*AA9A or with a truncated variant of the LPMO lacking the CBM. The CCOA/SEC/MALS analyses of the resulting cellulose fractions, subjected to this gradual dissolution **Approach I**, are presented in Table S2.

**Figure 4:**
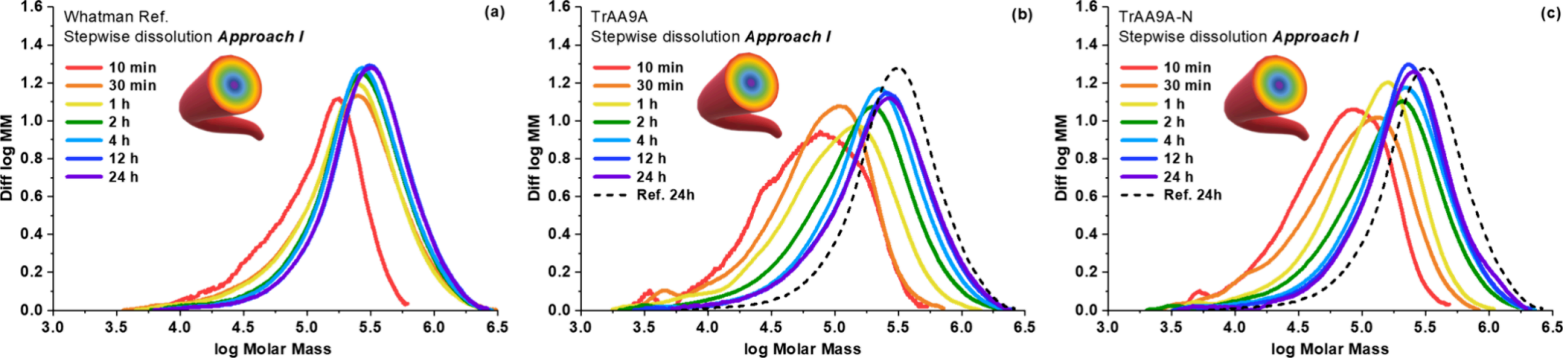
Molar mass distribution of LPMO-treated Whatman No. 1 fibers subjected to sequential dissolution in **Approach I**. The graphs show data for fibers solubilized after 10 min to 24 h of dissolution for untreated cellulose (a), cellulose treated with full-length *Tr*AA9A (b), and cellulose treated with truncated *Tr*AA9A-N (c). The dashed line in panels b and c equals the 24 h curve in panel (a). The LPMO reactions contained 500 mg of fibers, 0.064 μmol of LPMO per 1 g of dry fiber, and 1 mM GA in 50 mM sodium phosphate buffer, pH 7.0, in 20 mL of liquid volume and were incubated for 6 h at 45 °C. Note that the molar mass distributions are normalized to the same peak area and do not reflect the exact amount of material in each fraction.

Regarding the reference cellulose (untreated Whatman No. 1), only the shortest dissolution times yield a cellulose fraction with clearly shorter chains compared to those of the other fractions (Figure 4a). After the first 1 h of dissolution, lower molar mass material became less abundant, and the molar mass distribution in the subsequent samples (i.e., longer dissolution times) did not change significantly anymore. This indicates that a short chain fraction is present in a narrow outer layer of the untreated sample and makes up only a small part of the fiber mass, while the bulk material is rather homogeneous regarding the molar mass distribution. The molar mass distribution of the fully dissolved reference sample (after 24 h dissolution) features the narrow monomodal profile that is typical for cotton linters.

**Figure 5:**
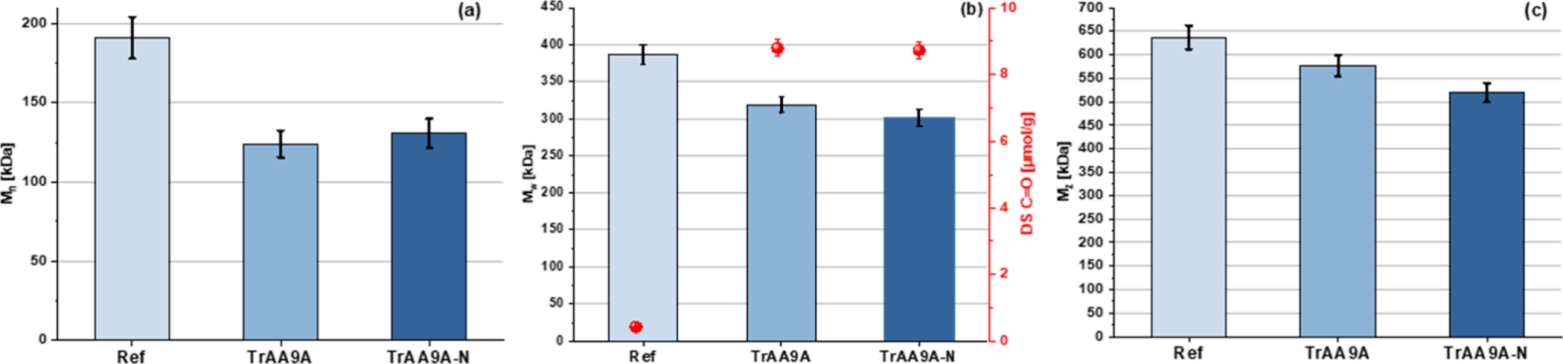
Properties of fibers dissolved with **Approach I**, after 24 h of dissolution. The graphs show the calculated statistical moments *M*_*n*_ (a), *M_w_* (b), and *M*_*z*_ (c) including experimental uncertainties for fibers generated in the reactions depicted in Figure 4, after dissolving these fibers for 24 h in **Approach I**. Validation data for experimental uncertainties are available in Table S1 and Figure S1. The middle graph additionally presents the carbonyl content (red dots) after dissolving the fibers for 24 h in **Approach I**, utilizing an RSD of 2.88% for C=O values based on Röhrling et al.^33^ The analyzed fractions contained both the outer and inner fiber layers. The graphs show an overall decrease of (a) *M*_*n*_, (b) *M*_*w*_, and (c) *M*_*z*_ for the fibers treated with *Tr*AA9A and *Tr*AA9A-N compared to the nontreated material. Data for all dissolution times are listed in Table S2.

Compared to the untreated cellulose, the molar mass distributions of both LPMO-treated celluloses showed a noticeable shift toward the lower molar mass range, indicative of the expected depolymerization of cellulose upon LPMO treatment (Figure 4b,c). The outer layers were affected more severely, as the shift was more prominent for shorter dissolution times (Figure S2 shows the corresponding molar mass distributions). On average, treatment of cellulose with both *Tr*AA9A variants, with and without CBM, resulted in a significant reduction in molar masses (*M*_*w*_ reduction for outer fiber layers exceeded 40% from the original value; the exact molar mass values for all fiber layers are given in Table S2) and in a 20-fold increase in the total amount of carbonyls in the outer fiber layers (Table S2). A closer look at the data for all dissolution times (Table S2) showed that, as expected, the average molar mass increases while the number of carbonyl groups decreases as the dissolution progresses and inner layers are also dissolved. Interestingly, while the LPMO-treated fibers had the same total carbonyl content (see 24 h dissolution samples depicted in Figure 5b), a closer look at the other dissolution fractions showed that the outermost layer of the *Tr*AA9A-treated fiber contained 50% more carbonyl groups as the outermost layer of the fiber treated with the truncated enzyme, *Tr*AA9A-N (Table S2). This indicates a CBM-dependent difference in fiber penetration and the mode of action of the LPMO. For example, this may suggest higher activity of *Tr*AA9A at the fiber surface while a greater tendency of *Tr*AA9A-N to penetrate deeper into the fiber core, as discussed in more detail in the next section.

The second dissolution approach (**Approach II**) is based on the complete isolation of the dissolved fraction from the nondissolved fibers after each dissolution period. Thus, **Approach II** allows for the characterization of distinct cellulose layers that are free from the (previously dissolved and removed) outer layers and from the not-yet-dissolved inner layers of the fibers (Figure 2). Although this approach is more tedious and time-consuming, it may provide additional information, for example, when evaluating the impact of an enzymatic treatment.

**Figure 6:**
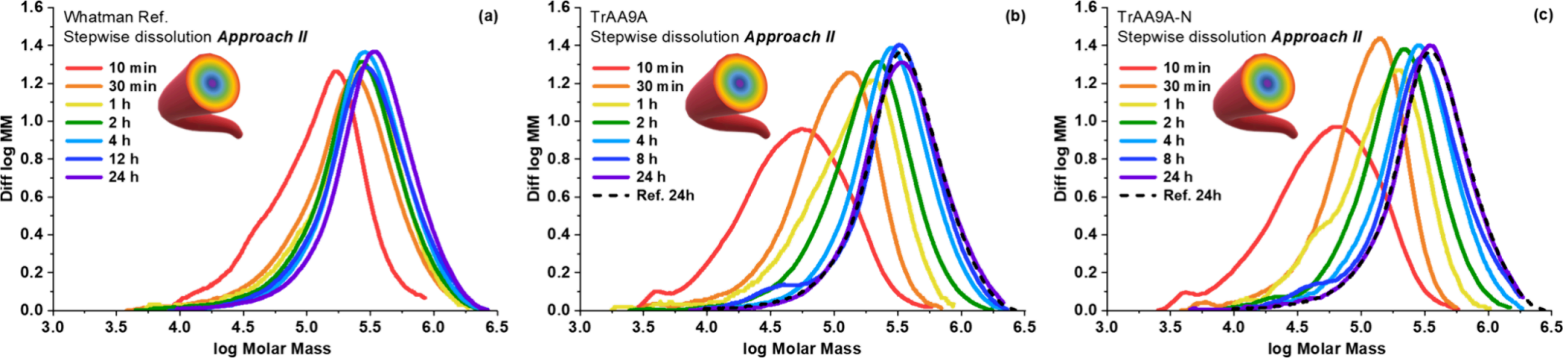
Molar mass distribution of LPMO-treated Whatman No. 1 fibers subjected to sequential dissolution of **Approach II**. The graphs show data for fibers solubilized after 10 min to 24 h of dissolution for untreated cellulose (a), cellulose treated with full-length *Tr*AA9A (b), and cellulose treated with truncated *Tr*AA9A-N (c). The dashed line in panels b and c equals the 24 h curve in panel (a). The LPMO reactions contained 500 mg of fibers, 0.064 μmol of LPMO per 1 g of dry fiber, and 1 mM GA in 50 mM sodium phosphate buffer, pH 7.0, in 20 mL liquid volume; and were incubated for 6 h at 45 °C. Note that the molar mass distributions are normalized to the same peak area and do not reflect the exact amount of material in each fraction.

**Figure 7:**
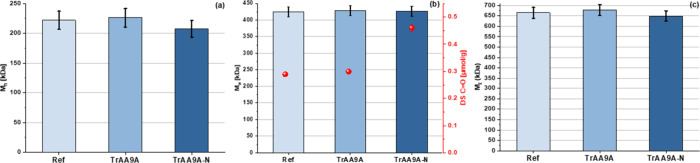
Properties of fibers dissolved with **Approach II**, after 24 h of dissolution. The graphs show the calculated statistical moments *M_n_* (a), *M_w_* (b), and *M*_*z*_ (c) including experimental uncertainties for fibers generated in the reactions depicted in Figure 6, after dissolving these fibers for 24 h in **Approach II**. Validation data for experimental uncertainties are available in Table S1 and Figure S1. The middle graph additionally presents the carbonyl content (red dots) after dissolving the fibers for 24 h in **Approach II**, utilizing an RSD of 2.88% for C=O values based on Röhrling et al.^33^ The analyzed fractions exclusively contained inner fiber layers. The graphs show an overall decrease of (a) *M*_*n*_, (b) *M*_*w*_, and (c) *M*_*z*_ for the fibers treated with *Tr*AA9A and *Tr*AA9A-N compared to the nontreated material. Data for all dissolution times are listed in Table S3.

Similarly to the stepwise dissolution with **Approach I**, the dissolution with filtration (**Approach II**) illustrates the presence of short-chain cellulosic molecules in the thin outermost fiber layer of untreated Whatman No. 1 fibers (Figure 6a). Subsequent samples corresponding to more inward layers of the cellulose yielded similar molar mass distribution profiles after 1–24 h, indicating a uniform molar mass distribution within the fibers. On the other hand, LPMO treatment of the cellulose led to a clear reduction in molar masses in fractions recovered after up to 4 h of dissolution with both *Tr*AA9A variants (Figure 6 b,c; Table S3) and to a prominent increase in the total number of carbonyl groups in nearly all fiber fractions (Table S3). As expected and as also shown by the results obtained with **Approach I** (Table S2), the outermost fiber layers were more affected than the underlying layers. In contrast to **Approach I**, the 24 h dissolution fraction for **Approach II** would contain only inner layers that are less likely to have been affected by the enzyme treatment. Accordingly, calculations of statistical moments for the 24 h fraction obtained with **Approach II**, showed no or only very minor effects of the enzyme treatment (Figure 7; note the contrast with Figure 5).

Importantly, the removal of the most affected outer fractions before further dissolution (**Approach II**) allowed us to observe a subtle enzyme-dependent variation in the innermost layers of the fiber. A slight decrease in molar mass data (*M*_*n*_ decreased from 221.7 to 207.5 kDa) and an overall increase in carbonyl groups (from 0.28 to 0.46 μmol/g) were only visible for the fibers treated with *Tr*AA9A-N (Figure 7), suggesting that this CBM-free enzyme was able to penetrate deeper toward the fiber core than the nontruncated *Tr*AA9A enzyme. Additional data show a similar trend for the other “late” dissolution times (4, 8 h; Table S3).

Comparing the two stepwise dissolution approaches, the less tedious **Approach I** can satisfactorily address the changes in molar mass and in total carbonyl content for the bulk sample and also provides insight how these parameters differ between the fiber surface and the fiber core. Collecting just two samples—at an early dissolution time point, corresponding to the outmost layers exclusively (i), and after completely dissolving the cellulose fibers, corresponding to the bulk sample including all fiber layers (ii)—would already provide valuable additional information on the fiber structure, compared to conventional single-step dissolution. In cases where higher resolution is desirable, for example, when assessing enzyme penetration into the fiber, **Approach II** is more suitable as it can reveal subtle changes in molar mass parameters for each fiber layer. Moreover, **Approach II** provides a detailed distribution of functional groups along the fiber cross-section. Whether these methods can provide the same level of information in the cross-sectional analysis of man-made fibers remains to be demonstrated, as the structure of rayon or lyocell fibers is very different from that of cotton. Nevertheless, both stepwise dissolution approaches should be capable of assessing basic surface-to-bulk differences in man-made fibers as well.

### Influence of the CBM on LPMO Action

The impact of CBMs on cellulose depolymerization has been studied extensively for cellulases, whereas less research has been done for LPMOs, as reviewed recently by Østby et al.^39^ It is universally acknowledged that CBMs enhance an enzyme’s binding affinity toward the substrate, and this has been shown convincingly several times for LPMOs as well.^9,11,12^ Some studies suggest that CBMs in cellulases may play a role in the amorphization of cellulose via the nonhydrolytic disruption of the hydrogen bonding in the crystalline areas.^40−42^ Such effects have not been described for CBMs appended to LPMOs and our present data, showing that the CBM containing LPMO penetrates less deeply into the fiber seem not compatible with a substrate-disrupting effect of the CBM. Regarding the catalytic performance of LPMOs, including oxidative regioselectivity, turnover rates, and redox stability, the impact of CBM1 is also not clear. This is due to a multitude of interwoven factors^13^ including (i) By affecting substrate affinity, the CBM also affects the resistance of the LPMO toward autocatalytic inactivation; (ii) In most studies, only soluble LPMO products are analyzed, whereas major, and potentially varying fraction, of oxidized sites (i.e., LPMO products) remains attached to the insoluble fiber.

Since it is generally not possible to unravel all these factors and since researchers perform their experiments in different ways, existing data for the impact of CBMs on LPMO action do not provide a clear and consistent picture. As an example, Chalak et al. reported an increase in the solubilization of C1-oxidized products for the CBM-free variant of C1/C4-oxidizing *Pa*LPMO9H from *Podospora anserina*, indicating an apparent change in regioselectivity after deleting the CBM.^12^ However, such a change in regioselectivity has not been reported for C1/C4-oxidizing *Tr*AA9A (also called *Hj*LPMO9A),^10^ a C1/C4-oxidizing bacterial LPMO,^43^ nor strictly C1- or C4-oxidizing LPMOs.^9,11^ As another example, several studies have reported that LPMO efficiency decreases after removal of the CBM,^44−46^ based on the detection of soluble oxidized products only and partly neglecting possible variation in LPMO autoinactivation. A more extended study by Courtade et al., who analyzed LPMO products in the soluble and insoluble fractions, has demonstrated that, similarly to cellulases,^47^ the impact of a CBM on the performance of LPMOs depends very much on the substrate concentration.^11^ Courtade et al. showed that the substrate concentration affects the CBM’s impact on overall substrate conversion, the ratio between soluble and insoluble oxidized products, and the resistance of the enzyme against oxidative damage, underpinning the complexity of the matter. In another study, Koskela et al. have shown that the naturally CBM-free *Nc*LPMO9F generates more carboxyl groups on the fibers and less solubilized oligosaccharides than CBM-containing *Nc*LPMO9E from *N. crassa*,^48^ much in line with the findings of Courtade et al.^11^ Both studies conclude that the anchoring of the LPMO to the substrate by a CBM leads to more localized oxidation (i.e., more cuts in the same region), increasing the chances of the same chain being cleaved twice, which is what is needed to release a soluble (= short) product. On the other hand, a CBM-free LPMO would cleave more randomly since it moves freely along the surface in between catalytic events. This conclusion is supported by a recent study that visualized oxidations on the fiber surface.^49^

**Figure 8:**
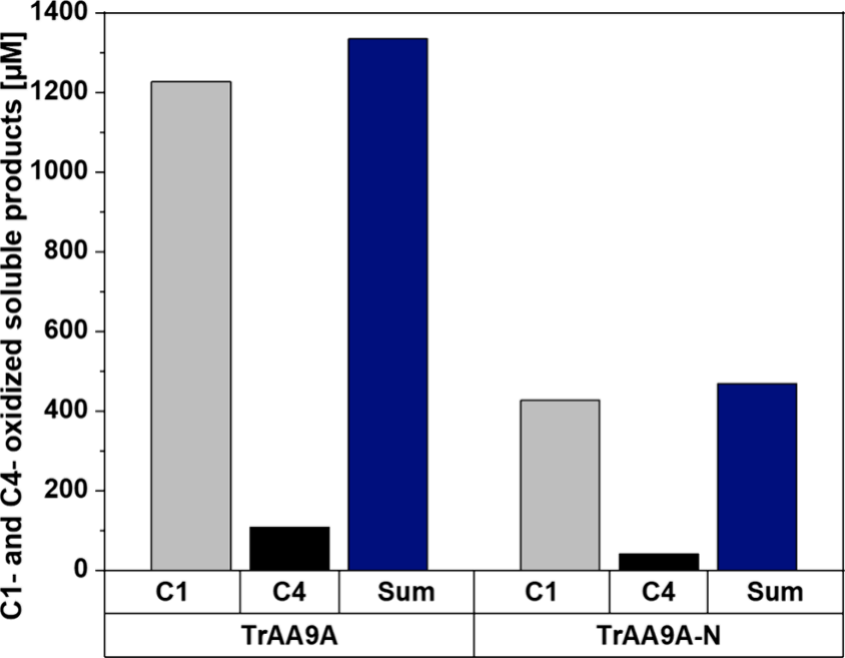
Generation of soluble oxidized products upon degradation of Whatman No. 1 fibers with full-length or truncated *Tr*AA9A. The LPMO reactions contained 500 mg of fibers, 0.064 μmol of LPMO per 1 g of dry fiber, and 1 mM GA in 50 mM sodium phosphate buffer, pH 7.0, in 20 mL liquid volume and were incubated for 6 h at 45 °C. Both C1- and C4-oxidized products were quantified after first reducing the complexity of the product mixture by treatment with a cellulase, as described in the Materials and Methods section.

The present results provide further insight into these matters. Analysis of fiber layers recovered by stepwise dissolution analysis consistently showed that fiber treatment with *Tr*AA9A (containing a CBM) leads to a more pronounced reduction in the *M*_*n*_ value than treatment with *Tr*AA9A-N without a CBM (Tables S1 and S2, Figure 5). On the other hand, *Tr*AA9A-N (lacking a CBM) reduced *M*_*w*_ and *M*_*z*_ to the same, or sometimes even higher extent compared to the full-length enzyme (Tables S1 and S2, Figure 5). The lower *M_n_* value and higher *M*_*w*_ and *M*_*z*_ values for the fiber fractions obtained with the CBM-carrying LPMO variant are compatible with a mode of action in which multiple oxidations happen close to each other on the fiber surface, leading to the production of relatively large amounts of water-soluble oligomeric fragments. Indeed, analysis of the soluble oxidized products showed that these were more abundantly produced by full-length *Tr*AA9A (Figure 8). Random cleavages at different positions on the surface affect predominantly the *M*_w_ and *M*_*z*_ values, as seen for the CBM-free variant.

Considering the ability of the LPMO variants to penetrate cellulose fibers and the extent of penetration, CBM-free LPMO variants are expected to be more capable of penetrating the fiber, because of their smaller size and higher mobility due to the lack of CBM-assisted substrate binding. The results obtained with **Approach II** (Figure 7, and Table S3) show that this indeed is the case: the statistical moments and carbonyl contents of the deeper fiber layers, i.e., samples after 4, 8, or 24 h dissolution consistently show that the material treated with *Tr*AA9A-N has a lower average mass and higher carbonyl content compared to the material treated with the full-length *Tr*AA9A. Apparently, the CBM-free variant was able to reach the fiber core through the cracks in the outer fiber layer, although its overall impact on the fiber interior remained low.

**Figure 9:**
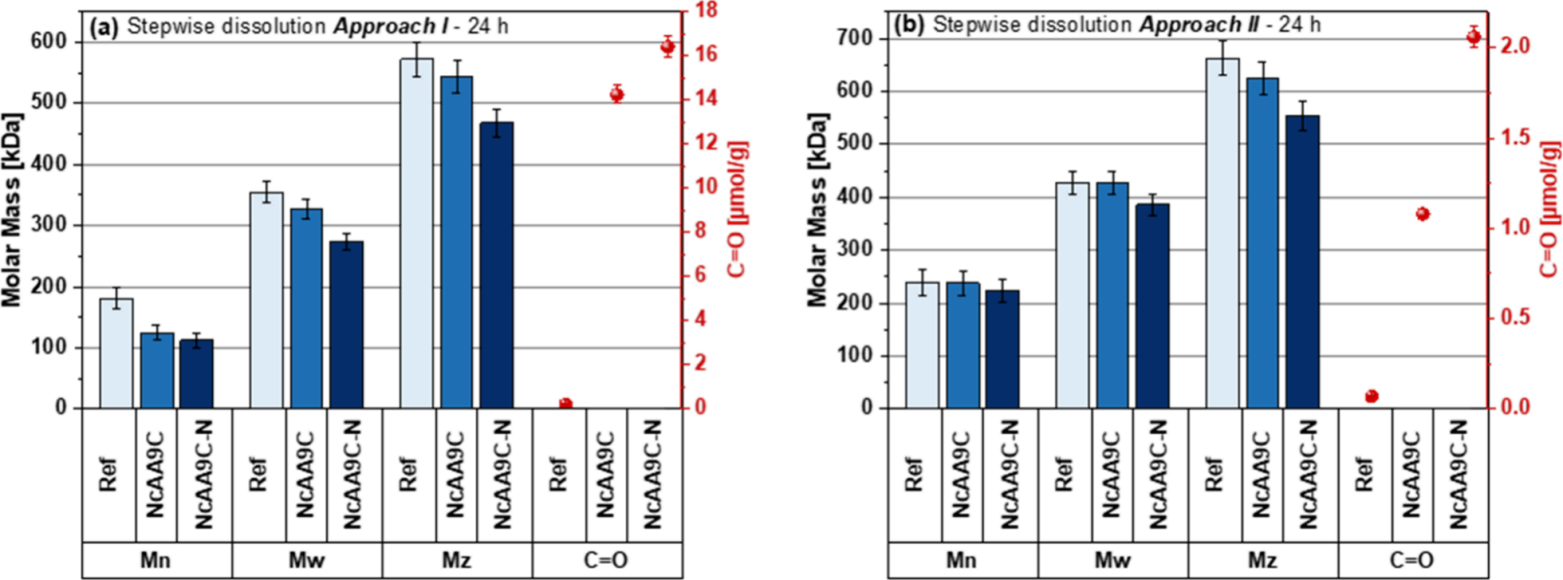
Changes in Whatman No. 1 fibers upon treatment with *Nc*AA9C or *Nc*AA9C–N. The figure shows the calculated average molar masses and carbonyl content including experimental uncertainties for the last sample (24 h) obtained after gradual dissolution, using (a) **Approach I** or (b) **Approach II**. When using these approaches, the 24 h dissolution samples represent the bulk material, including all fiber layers and the innermost fiber core fraction, respectively. The LPMO reactions contained 50 mg of fibers, 0.05 μmol of LPMO per 1 g of dry fiber, and 1 mM GA in 50 mM Bis-Tris/HCl buffer, pH 6.5, in 5 mL liquid volume and were incubated for 24 h at 30 °C. Estimations of experimental uncertainties are provided in Table S1 and Figure S1.

To obtain additional insight into these CBM effects and to further validate the newly developed sequential dissolution methods, Whatman No. 1 fibers were treated with C4-oxidizing *Nc*AA9C and its truncated, CBM-free variant, *Nc*AA9C–N. The 24 h dissolution samples (Figure 9), show the same trends as seen with *Tr*AA9A (Figures 5 and 7): the truncated enzyme has a bigger effect on the inner layers of the fiber, with a relatively higher reduction in molar mass and higher insertion of carbonyl groups. Figure 9 also nicely illustrates a key difference between the two sample dissolution approaches: **Approach I** yields similar carbonyl contents for the two LPMO treatments in the 24 h dissolution sample, which is not surprising since with this method the 24 h dissolution sample contains all fibers (Figure 9a). On the other hand, **Approach II** shows a distinct difference in the carbonyl content of the innermost layer for the two LPMO forms, underpinning the different mechanisms of action of the two *Nc*AA9C variants (Figure 9b). Taken together, the data for *Tr*AA9A and *Nc*AA9C show that the CBM-free LPMO has a stronger effect on the deeper layers of the cellulose fiber.

## Conclusions

Evaluating how enzymatic or chemical treatments affect fiber structure and whether these changes are limited to the surface or impact the entire fiber is crucial for assessing treatment efficiency and the extent of modification in cellulose engineering. In this study, we compared two stepwise dissolution approaches that enabled us to assess modifications throughout the entire depth of the cellulose fiber. The first approach (**Approach I**), which does not require sample filtration and thus is easier to implement, addresses the changes in molar mass distribution and total carbonyl content between the outer fiber layer(s) and the bulk material. The second approach (**Approach II**), although more tedious and time-consuming due to the separation of the dissolved fractions by filtration after each dissolution step, provides better insight into the characteristics of a few distinct fiber layers including the distribution of functional groups and changes in molar mass parameters along the cross-section.

Here, we demonstrate the applicability of these stepwise dissolution methods for analyzing Whatman No. 1 cellulose with LPMO enzymes. The stepwise dissolution approach revealed the depth of enzymatic activity within the fibers and highlighted the different mechanisms at play for CBM-carrying and CBM-free LPMOs. Furthermore, our findings underscore the significance of characterizing cellulose properties in conjunction with the detection of soluble products to estimate the efficiency of LPMOs. Importantly, the cross-sectional fiber analyses established here are not limited to a specific fiber type or to enzyme-driven modifications and will be expanded for examining other fiber modifications, where spatial resolution is a key factor in future research.

## Supporting Information Available

Calculation and statistical evaluation of experimental uncertainties for molar mass parameters determined by size exclusion chromatography; calculated statistical moments for Whatman No. 1 fibers before and after treatment with *Tr*AA9A (with CBM) and *Tr*AA9A-N (without CBM) obtained using stepwise dissolution with **Approach I** and **Approach II**; molar mass distribution of outer layers of Whatman No. 1 fibers solubilized after 10 min of dissolution before and after treatment with *Tr*AA9A (with CBM) and *Tr*AA9A-N (without CBM), showing degradation of the outer fiber layers after enzymatic treatment (PDF)

## References

[ref1] Mussatto S.; van Loosdrecht M. Cellulose: a key polymer for a greener, healthier, and bio-based future. Biofuel Res. J. 2016, 12, 482 10.18331/BRJ2016.3.4.2.

[ref2] Lopes A. M.; Ferreira Filho E. X.; Moreira L. R. S. An update on enzymatic cocktails for lignocellulose breakdown. J. Appl. Microbiol. 2018, 125, 632–645. 10.1111/jam.13923. 29786939

[ref3] Chylenski P.; Bissaro B.; So̷rlie M.; Ro̷hr Å.K.; Várnai A.; Horn S. J.; Eijsink V. G. H. Lytic polysaccharide monooxygenases in enzymatic processing of lignocellulosic biomass. ACS Catal. 2019, 9 (6), 4970–4991. 10.1021/acscatal.9b00246.

[ref4] Arantes V.; Dias I. K. R.; Berto G. L.; Pereira B.; Marotti B. S.; Nogueira C. F. O. The current status of the enzyme-mediated isolation and functionalization of nanocelluloses: production, properties, techno-economics, and opportunities. Cellulose 2020, 27, 10571–10630. 10.1007/s10570-020-03332-1.

[ref5] Tõlgo M.; Hegnar O. A.; Østby H.; Várnai A.; Vilaplana F.; Eijsink V. G. H.; Olsson L. Comparison of six lytic polysaccharide monooxygenases from *Thermothielavioides terrestris* shows that functional variation underlies the multiplicity of LPMO genes in filamentous fungi. Appl. Environ. Microbiol. 2022, 88 (6), e0009622 10.1128/aem.00096-22. 35080911 PMC8939357

[ref6] Karnaouri A.; Chorozian K.; Zouraris D.; Karantonis A.; Topakas E.; Rova U.; Christakopoulos P. Lytic polysaccharide monooxygenases as powerful tools in enzymatically assisted preparation of nano-scaled cellulose from lignocellulose: A review. Bioresour. Technol. 2022, 345, 126491 10.1016/j.biortech.2021.126491. 34871721

[ref7] Vaaje-Kolstad G.; Forsberg Z.; Loose J. S. M.; Bissaro B.; Eijsink V. G. H. Structural diversity of lytic polysaccharide monooxygenases. Curr. Opin. Struct. Biol. 2017, 44, 67–76. 10.1016/j.sbi.2016.12.012. 28086105

[ref8] Vaaje-Kolstad G.; Westereng B.; Horn S. J. H.; Liu Z.; Zhai H.; So̷rlie M.; Eijsink V. G. H. An oxidative enzyme boosting the enzymatic conversion of recalcitrant polysaccharides. Science 2010, 330 (6001), 219–222. 10.1126/science.1192231. 20929773

[ref9] Laurent C. V. F. P.; Sun P.; Scheiblbrandner S.; Csarman F.; Cannazza P.; Frommhagen M.; van Berkel W. J. H.; Oostenbrink C.; Kabel M. A.; Ludwig R. Influence of lytic polysaccharide monooxygenase active site segments on activity and affinity. Int. J. Mol. Sci. 2019, 20 (24), 6219 10.3390/ijms20246219. 31835532 PMC6940765

[ref10] Danneels B.; Tanghe M.; Desmet T. Structural features on the substrate-binding surface of fungal lytic polysaccharide monooxygenases determine their oxidative regioselectivity. Biotechnol. J. 2019, 14 (3), e1800211 10.1002/biot.201800211. 30238672

[ref11] Courtade G.; Forsberg Z.; Heggset E. B.; Eijsink V. G. H.; Aachmann F. L. The carbohydrate-binding module and linker of a modular lytic polysaccharide monooxygenase promote localized cellulose oxidation. J. Biol. Chem. 2018, 293 (34), 13006–13015. 10.1074/jbc.RA118.004269. 29967065 PMC6109919

[ref12] Chalak A.; Villares A.; Moreau C.; Haon M.; Grisel S.; d’Orlando A.; Herpoël-Gimbert I.; Labourel A.; Cathala B.; Berrin G.-J. Influence of the carbohydrate-binding module on the activity of a fungal AA9 lytic polysaccharide monooxygenase on cellulosic substrates. Biotechnol. Biofuels 2019, 12, 206 10.1186/s13068-019-1548-y. 31508147 PMC6721207

[ref13] Forsberg Z.; Courtade G. On the impact of carbohydrate-binding modules (CBMs) in lytic polysaccharide monooxygenases (LPMOs). Essays Biochem. 2023, 67 (3), 561–574. 10.1042/EBC20220162. 36504118 PMC10154629

[ref14] Song B.; Li B.; Wang X.; Shen W.; Park S.; Collings C.; Feng A.; Smith S. J.; Walton J. D.; Ding S.-Y. Real-time imaging reveals that lytic polysaccharide monooxygenase promotes cellulase activity by increasing cellulose accessibility. Biotechnol. Biofuels 2018, 11, 41 10.1186/s13068-018-1023-1. 29467819 PMC5815216

[ref15] Heijnesson-Hultén A. Kraft pulp fibre surfaces: Chemical composition and effect on pulp bleachability; Ph.D Dissertation, Chalmers University of Technology: Göteborg, Sweeden, 1996.

[ref16] Sjöberg J.; Kleen M.; Dahlman O.; Agnemo R. Analyses of carbohydrates and lignin in the surface and inner layers of softwood pulp fibers obtained employing various alkaline cooking processes. Nord. Pulp Pap. Res. J. 2002, 17 (3), 295–301. 10.3183/npprj-2002-17-03-p295-301.

[ref17] Heijnesson A.; Simonson R.; Westermark U. Removal of lignin-rich surface material from unbleached kraft fibres. Holzforschung 1995, 49 (4), 313 10.1515/hfsg.1995.49.4.313.

[ref18] Sjöberg J.; Potthast A.; Rosenau T.; Kosma P.; Sixta H. Cross-sectional analysis of the polysaccharide composition in cellulosic fiber materials by enzymatic peeling/high-performance capillary zone electrophoresis. Biomacromolecules 2005, 6 (6), 3146–3151. 10.1021/bm050471j. 16283739

[ref19] Tenkanen M.; Hausalo T.; Siika-Aho M.; Buchert J.; Viikari L. Use of enzymes in combination with anion exchange chromatography in the analysis of carbohydrate composition of kraft. In Proceedings of the 8th International Symposium on Wood and Pulping Chemistry; Helsinki, Finland, June 6–9, 1995.

[ref20] Luce J. Radial distribution of properties through the cell wall. Pulp Pap. Mag. Can. 1964, 65 (10), T419–T423.

[ref21] Henniges U.; Kostic M.; Borgards A.; Rosenau T.; Potthast A. Dissolution behavior of different celluloses. Biomacromolecules 2011, 12, 871–879. 10.1021/bm101555q. 21391580

[ref22] Kallmes O. The distribution of constituents across the wall of unbleached spruce sulfite fibers. Tappi J. 1959, 43 (2), 143–152.

[ref23] Sulaeva I.; Budischowsky D.; Rahikainen J.; Marjamaa K.; Sto̷pamo F.; Khaliliyan H.; Melikhov I.; Rosenau T.; Kruus K.; Várnai A.; Eijsink V. G. H.; Potthast A. A novel approach to analyze the impact of lytic polysaccharide monooxygenases (LPMOs) on cellulosic fibres. Carbohydr. Polym. 2024, 328, 121696 10.1016/j.carbpol.2023.121696. 38220335

[ref24] Drula E.; Garron M.-L.; Dogan S.; Lombard V.; Henrissat B.; Terrapon N. The carbohydrate-active enzyme database: functions and literature. Nucleic Acids Res. 2022, 50 (D1), D571–D577. 10.1093/nar/gkab1045. 34850161 PMC8728194

[ref25] Kont R.; Pihlajaniemi V.; Borisova A. S.; Aro N.; Marjamaa K.; Loogen J.; Büchs J.; Eijsink V. G. H.; Kruus K.; Väljamäe P. The liquid fraction from hydrothermal pretreatment of wheat straw provides lytic polysaccharide monooxygenases with both electrons and H_2_O_2_ co-substrate. Biotechnol. Biofuels 2019, 12, 235 10.1186/s13068-019-1578-5. 31624497 PMC6781412

[ref26] Kittl R.; Kracher D.; Burgstaller D.; Haltrich D.; Ludwig R. Production of four *Neurospora crassa* lytic polysaccharide monooxygenases in Pichia pastoris monitored by a fluorimetric assay. Biotechnol. Biofuels 2012, 5, 79 10.1186/1754-6834-5-79. 23102010 PMC3500269

[ref27] Borisova A. S.; Isaksen T.; Dimarogona M.; Kognole A. A.; Mathiesen G.; Várnai A.; Ro̷hr Å.K.; Payne C. M.; So̷rlie M.; Sandgren M.; Eijsink V. G. H. Structural and functional characterization of a lytic polysaccharide monooxygenase with broad substrate specificity. J. Biol. Chem. 2015, 290 (38), 22955–22969. 10.1074/jbc.M115.660183. 26178376 PMC4645601

[ref28] Ståhlberg J.; Divne C.; Koivula A.; Piens K.; Claeyssens M.; Teeri T. T.; Jones T. A. Activity studies and crystal structures of catalytically deficient mutants of cellobiohydrolase I from *Trichoderma reesei*. J. Mol. Biol. 1996, 264 (2), 337–349. 10.1006/jmbi.1996.0644. 8951380

[ref29] Zámocký M.; Schümann C.; Sygmund C.; O’Callaghan J.; Dobson A. D. W.; Ludwig R.; Haltrich D.; Peterbauer C. K. Cloning, sequence analysis and heterologous expression in *Pichia pastoris* of a gene encoding a thermostable cellobiose dehydrogenase from *Myriococcum thermophilum*. Protein Expr. Purif. 2008, 59 (2), 258–265. 10.1016/j.pep.2008.02.007. 18374601

[ref30] Marjamaa K.; Rahikainen J.; Karjalainen M.; Maiorova N.; Holopainen-Mantila U.; Molinier M.; Aro N.; Nygren H.; Mikkelson A.; Koivula A.; Kruus K. Oxidative modification of cellulosic fibres by lytic polysaccharide monooxygenase AA9A from *Trichoderma reesei*. Cellulose 2022, 29, 6021–6038. 10.1007/s10570-022-04648-w.

[ref31] Tuveng T. R.; Jensen M. S.; Fredriksen L.; Vaaje-Kolstad G.; Eijsink V. G. H.; Forsberg Z. A thermostable bacterial lytic polysaccharide monooxygenase with high operational stability in a wide temperature range. Biotechnol. Biofuels 2020, 13 (1), 194 10.1186/s13068-020-01834-5. 33292445 PMC7708162

[ref32] Müller G.; Várnai A.; Johansen K. S.; Eijsink V. G. H.; Horn S. J. Harnessing the potential of LPMO-containing cellulase cocktails poses new demands on processing conditions. Biotechnol. Biofuels 2015, 8, 187 10.1186/s13068-015-0376-y. 26609322 PMC4659242

[ref33] Röhrling J.; Potthast A.; Rosenau T.; Lange T.; Ebner G.; Sixta H.; Kosma P. A novel method for the determination of carbonyl groups in cellulosics by fluorescence labeling. 1. Method Development. Biomacromolecules 2002, 3 (5), 959–968. 10.1021/bm020029q. 12217041

[ref34] Zhang C.; Liu R.; Xiang J.; Kang H.; Liu Z.; Huang Y. Dissolution mechanism of cellulose in *N*,*N*-dimethylacetamide/lithium chloride: revisiting through molecular interactions. J. Phys. Chem. B 2014, 118 (31), 9507–9514. 10.1021/jp506013c. 25026263

[ref35] Sen S.; Martin J. D.; Argyropoulos D. S. Review of cellulose non-derivatizing solvent interactions with emphasis on activity in inorganic molten salt hydrates. ACS Sustain. Chem. Eng. 2013, 1 (8), 858–870. 10.1021/sc400085a.

[ref36] Rebiere J.; Heuls M.; Castignolles P.; Gaborieau M.; Rouilly A.; Violleau F.; Durrieu V. Structural modifications of cellulose samples after dissolution into various solvent systems. Anal. Bioanal. Chem. 2016, 408 (29), 8403–8414. 10.1007/s00216-016-9958-1. 27695986

[ref37] Pionteck H.; Berger W.; Morgenstern B.; Fengel D. Changes in cellulose structure during dissolution in LiCl:*N*,*N*-dimethylacetamide and in the alkaline iron tartrate system EWNN. Cellulose 1996, 3, 127–139. 10.1007/BF02228796.

[ref38] Jonoobi M.; Oladi R.; Davoudpour Y.; Oksman K.; Dufresne A.; Hamzeh Y.; Davoodi R. Different preparation methods and properties of nanostructured cellulose from various natural resources and residues: a review. Cellulose 2015, 22, 935–969. 10.1007/s10570-015-0551-0.

[ref39] Østby H.; Hansen L. D.; Horn S. J.; Eijsink V. G. H.; Várnai A. Enzymatic processing of lignocellulosic biomass: principles, recent advances and perspectives. J. Ind. Microbiol. Biotechnol. 2020, 47, 623–657. 10.1007/s10295-020-02301-8. 32840713 PMC7658087

[ref40] Bernardes A.; Pellegrini V. O. A.; Curtolo F.; Camilo C. M.; Mello B. L.; Johns M. A.; Scott J. L.; Guimaraes F. E. C.; Polikarpov I. Carbohydrate binding modules enhance cellulose enzymatic hydrolysis by increasing access of cellulases to the substrate. Carbohydr. Polym. 2019, 211, 57–68. 10.1016/j.carbpol.2019.01.108. 30824104

[ref41] Arantes V.; Saddler J. N. Access to cellulose limits the efficiency of enzymatic hydrolysis: the role of amorphogenesis. Biotechnol. Biofuels 2010, 3, 4 10.1186/1754-6834-3-4. 20178562 PMC2844368

[ref42] Banka R.; Mishra S.; Ghose T. Fibril formation from cellulose by a novel protein from *Trichoderma reesei*: a non-hydrolytic cellulolytic component. World. J. Microbiol. Biotechnol. 1998, 14 (4), 551–558. 10.1023/A:1008888331936.

[ref43] Forsberg Z.; Bissaro B.; Gullesen J.; Dalhus B.; Vaaje-Kolstad G.; Eijsink V. G. H. Structural determinants of bacterial lytic polysaccharide monooxygenase functionality. J. Biol. Chem. 2018, 293 (4), 1397–1412. 10.1074/jbc.M117.817130. 29222333 PMC5787815

[ref44] Crouch L.; Labourel A.; Walton P. H.; Davies G. J.; Gilbert H. J. The Contribution of non-catalytic carbohydrate binding modules to the activity of lytic polysaccharide monooxygenases. J. Biol. Chem. 2016, 291 (14), 7439–7449. 10.1074/jbc.M115.702365. 26801613 PMC4817175

[ref45] Forsberg Z.; Mackenzie A. K.; So̷rlie M.; Ro̷hr Å.K.; Helland R.; Arvai A. S.; Vaaje-Kolstad G.; Eijsink V. G. H. Structural and functional characterization of a conserved pair of bacterial cellulose-oxidizing lytic polysaccharide monooxygenases. Proc. Natl. Acad. Sci. U. S. A. 2014, 111 (23), 8446–8451. 10.1073/pnas.1402771111. 24912171 PMC4060697

[ref46] Arfi Y.; Shamshoum M.; Rogachev I.; Peleg Y.; Bayer E. A. Integration of bacterial lytic polysaccharide monooxygenases into designer cellulosomes promotes enhanced cellulose degradation. Proc. Natl. Acad. Sci. U.S.A. 2014, 111 (25), 9109–9114. 10.1073/pnas.1404148111. 24927597 PMC4078869

[ref47] Várnai A.; Siika-aho M.; Viikari L. Carbohydrate-binding modules (CBMs) revisited: reduced amount of water counterbalances the need for CBMs. Biotechnol. Biofuels 2013, 6, 30 10.1186/1754-6834-6-30. 23442543 PMC3599012

[ref48] Koskela S.; Wang S.; Xu D.; Yang X.; Li K.; Berglund L. A.; McKee L. S.; Bulone V.; Zhou Q. Lytic polysaccharide monooxygenase (LPMO) mediated production of ultra-fine cellulose nanofibres from delignified softwood fibres. Green Chem. 2019, 21, 5924–5933. 10.1039/C9GC02808K.

[ref49] Raji O.; Eijsink V. G. H.; Master E.; Forsberg Z. Modularity impacts cellulose surface oxidation by a lytic polysaccharide monooxygenase from *Streptomyces coelicolor*. Cellulose 2023, 30, 10783–10794. 10.1007/s10570-023-05551-8.

