# Supplementary material for: Beyond the Surface: A Methodological Exploration of Enzyme Impact along the Cellulose Fiber Cross-Section
Source: Biomacromolecules. 2024 Apr 18;25(5):3076–86. doi: 10.1021/acs.biomac.4c00152 (PMC11094719; doi:10.1021/acs.biomac.4c00152)
Supplement: Supplementary file 1 — bm4c00152_si_001.pdf [file bm4c00152_si_001.pdf]

Supporting information

for

Beyond the Surface: A Methodological Exploration of Enzyme Impact along the Cellulose Fiber Cross-Section

*Irina Sulaeva[1], Fredrik Gjerstad Støpamo[2], Ivan Melikhov[3], David Budischowsky[3], Jenni L. Rahikainen[4], Anna Borisova[4], Kaisa Marjamaa[4], Kristiina Kruus[4,5], Vincent G.H. Eijsink[2], Anikó Várnai[2], Antje Potthast[3]**

[1] Core Facility Analysis of Lignocellulosics (ALICE), University of Natural Resources and Life Sciences, Vienna (BOKU), Konrad Lorenz-Straße 24, A-3430 Tulln an der Donau, Austria

[2] Faculty of Chemistry, Biotechnology and Food Science, Norwegian University of Life Sciences, 1432, Ås, Norway

[3] Institute of Chemistry of Renewable Resources, Department of Chemistry, University of Natural Resources and Life Sciences, Vienna (BOKU), Konrad Lorenz-Straße 24, A-3430 Tulln an der Donau, Austria

[4] Solutions for Natural Resources and Environment, VTT Technical Research Centre of Finland Ltd, Tietotie 2, FI-02044, Espoo, Finland

[5] School of Chemical Engineering, Aalto University, P.O. Box 16100, Espoo, 00076 AALTO, Finland

* Corresponding author: antje.potthast@boku.ac.at

Table S1. Calculated statistical moments for untreated Whatman No. 1 reference fibers obtained in ten independent measurements. The table shows the average values for each parameter, as well as standard deviations and relative standard deviations (RSD), providing an estimation of experimental uncertainties for measured parameters.

**Sample**	**M_n (kDa)**	**M_w (kDa)**	**M_z (kDa)**
Wh_Ref_1	191.0	386.4	637.0
Wh_Ref_2	193.8	386.6	641.8
Wh_Ref_3	194.2	392.2	632.6
Wh_Ref_4	182.8	378.8	624.5
Wh_Ref_5	177.9	386.3	628.7
Wh_Ref_6	201.0	394.1	614.0
Wh_Ref_7	181.0	355.2	572.5
Wh_Ref_8	215.5	388.5	595.2
Wh_Ref_9	210.5	384.0	588.5
Wh_Ref_10	178.3	359.0	592.1
**Average value**	192.6	381.11	612.69
**Standard deviation**	13.22	13.36	23.95
**RSD, %**	**6.86**	**3.50**	**3.91**

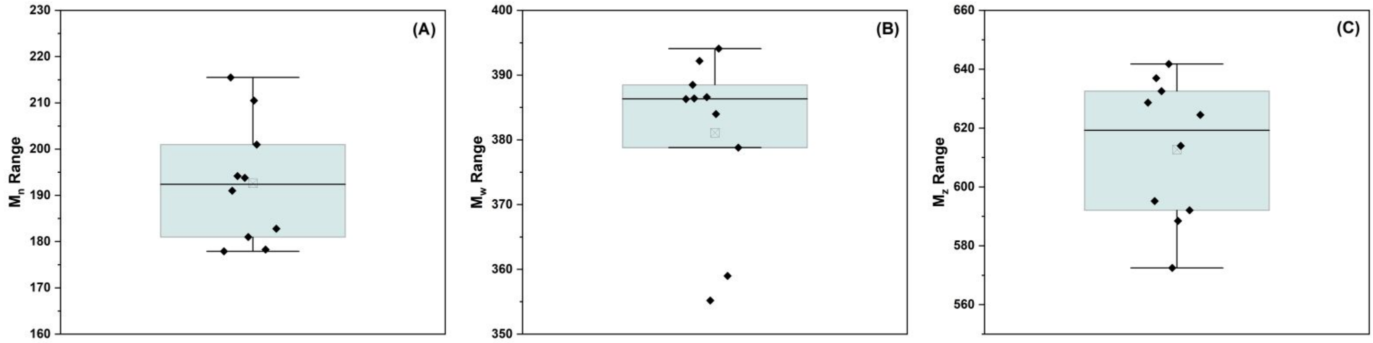

Figure S1. Statistical evaluation of experimental uncertainties based on ten independent measurements of untreated Whatman No. 1 reference fibers. The box plots demonstrate the spread of M_n (panel A), M_w (panel B), and M_z (panel C) values. The diamonds show the results for individual measurements; the empty squares show the mean of the data; the boxes are drawn

from Q1 to Q3 with a horizontal line denoting the median; the whiskers are drawn within the 1.5 IQR value.

Table S2. Calculated statistical moments for Whatman No. 1 fibers including experimental uncertainties before and after treatment with *Tr*AA9A (with CBM) and *Tr*AA9A-N (without CBM) obtained using stepwise dissolution with **Approach I**. The LPMO reactions contained 500 mg fibers, 0.064 µmol LPMO per 1 g of dry fiber, and 1 mM GA in 50 mM sodium phosphate buffer, pH 7.0, in 20 mL liquid volume and were incubated for 6 h at 45 °C.

Sample	Dissolution time	M_n (kDa)	M_w (kDa)	M_z (kDa)	Đ	C=O total (µmol/g)
Untreated	10 min	75.81 ± 5.20	149.5 ± 5.23	218.5 ± 8.54	1.97	5.99 ± 0.18
	30 min	83.87 ± 5.75	261.6 ± 9.16	532.1 ± 20.81	3.12	3.65 ± 0.11
	1 h	119.5 ± 8.20	299.5 ± 10.48	544.6 ± 21.29	2.51	2.52 ± 0.08
	2 h	154.6 ± 10.61	341.0 ± 11.94	582.4 ± 22.77	2.21	1.32 ± 0.04
	4 h	169.3 ± 11.61	351.2 ± 12.29	597.6 ± 23.37	2.07	0.77 ± 0.03
	12 h	192.9 ± 13.23	389.5 ± 13.63	636.2 ± 24.88	2.02	0.39 ± 0.02
	24 h	191.0 ± 13.10	386.4 ± 13.52	637.0 ± 24.91	2.02	0.43 ± 0.02
*Tr*AA9A-treated	10 min	35.26 ± 2.42	89.14 ± 3.12	154.6 ± 6.04	2.53	73.53 ± 2.12
	30 min	38.84 ± 2.66	101.2 ± 3.54	172.8 ± 6.76	2.61	35.27 ± 1.02
	1 h	48.11 ± 3.30	150.7 ± 5.27	289.2 ± 11.31	3.13	28.43 ± 0.82
	2 h	72.84 ± 5.00	221.2 ± 7.74	425.1 ± 16.62	3.04	17.05 ± 0.5
	4 h	103.5 ± 7.10	270.9 ± 9.48	488.4 ± 19.10	2.62	11.61 ± 0.34
	12 h	116.8 ± 8.01	310.6 ± 10.87	559.7 ± 21.88	2.66	9.11 ± 0.27
	24 h	124.0 ± 8.51	319.7 ± 11.19	577.6 ± 22.58	2.58	8.79 ± 0.26
*Tr*AA9A-N-treated	10 min	39.97 ± 2.74	87.59 ± 3.07	142.1 ± 5.56	2.19	49.18 ± 1.42
	30 min	44.84 ± 3.08	119.0 ± 4.17	207.9 ± 8.13	2.65	33.46 ± 0.97
	1 h	65.94 ± 4.52	154.7 ± 5.41	253.6 ± 9.92	2.35	23.27 ± 0.68
	2 h	79.14 ± 5.43	227.7 ± 7.97	416.0 ± 16.27	2.88	15.42 ± 0.45
	4 h	103.3 ± 7.09	265.6 ± 9.30	470.3 ± 18.39	2.57	10.58 ± 0.31
	12 h	121.9 ± 8.36	281.2 ± 9.84	481.3 ± 18.82	2.31	8.57 ± 0.25
	24 h	130.8 ± 8.97	302.0 ± 10.57	519.9 ± 20.33	2.31	8.73 ± 0.26

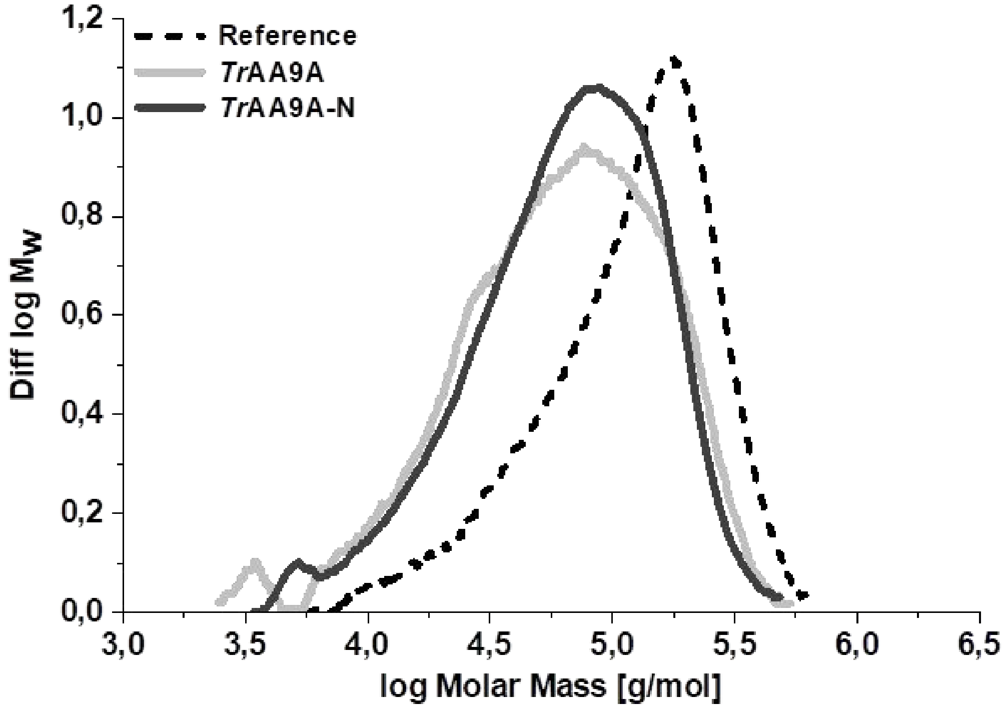

Figure S2. Molar mass distribution of outer layer of Whatman No. 1 fibers solubilized after 10 min of dissolution for untreated cellulose (dashed line), cellulose treated with full-length *Tr*AA9A (light grey) and cellulose treated with truncated *Tr*AA9A-N (dark grey), demonstrating pronounced degradation of the outer fibre layers occuring upon enzymatic treatment. The LPMO reactions contained 500 mg fibers, 0.064 µmol LPMO per 1 g of dry fiber, and 1 mM GA in 50 mM sodium phosphate buffer, pH 7.0, in 20 mL liquid volume and were incubated for 6 h at 45 ˚C. Note that the molar mass distributions are normalized to the same peak area and do not reflect the exact amount of material in each fraction.

Table S3. Calculated statistical moments for Whatman No. 1 fibres including experimental uncertainties before and after treatment with *Tr*AA9A (with CBM) and *Tr*AA9A-N (without CBM) obtained using stepwise dissolution with **Approach II**. The LPMO reactions contained 500 mg fibers, 0.064 μmol LPMO per 1 g of dry fiber, and 1 mM GA in 50 mM sodium phosphate buffer, pH 7.0, in 20 mL liquid volume and were incubated for 6 h at 45 °C.

**Sample**	**Dissolution time**	**M_n (kDa)**	**M_w (kDa)**	**M_z (kDa)**	**Đ**	**C=O total (μmol/g)**
Untreated	0-10 min	77.84 ± 5.34	158.9 ± 5.57	259.5 ± 10.15	2.04	8.05 ± 0.24
	10-30 min	117.1 ± 8.04	283.6 ± 9.93	490.3 ± 19.18	2.42	1.99 ± 0.06
	30-60 min	132.9 ± 9.12	312.8 ± 10.95	504.0 ± 19.71	2.35	1.81 ± 0.06
	1-2 h	152.3 ± 10.45	331.1 ± 11.59	548.7 ± 21.46	2.17	0.45 ± 0.02
	2-4 h	201.9 ± 13.86	372.0 ± 13.02	593.5 ± 23.21	1.84	0.24 ± 0.01
	4-8 h	196.5 ± 13.48	383.9 ± 13.44	642.4 ± 25.12	1.95	0.29 ± 0.01
	8-24 h	221.7 ± 15.21	423.9 ± 14.84	664.8 ± 26.00	1.91	0.28 ± 0.01
*Tr*AA9A-treated	0-10 min	28.59 ± 1.97	69.75 ± 2.45	133.2 ± 5.21	2.44	58.02 ± 1.68
	10-30 min	59.24 ± 4.07	123.4 ± 4.32	187.7 ± 7.34	2.08	21.31 ± 0.62
	30-60 min	68.65 ± 4.71	177.6 ± 6.22	275.7 ± 10.78	2.59	13.63 ± 0.4
	1-2 h	118.2 ± 8.11	243.8 ± 8.54	393.2 ± 15.38	2.06	5.17 ± 0.15
	2-4 h	183.2 ± 12.57	344.2 ± 12.05	535.9 ± 20.96	1.88	1.52 ± 0.05
	4-8 h	178.4 ± 12.24	388.9 ± 13.62	611.9 ± 23.93	2.18	1.06 ± 0.04
	8-24 h	226.0 ± 15.51	429.0 ± 15.02	678.0 ± 26.51	1.90	0.30 ± 0.01
*Tr*AA9A-N-treated	0-10 min	31.01 ± 2.13	75.54 ± 2.65	137.4 ± 5.38	2.44	56.72 ± 1.64
	10-30 min	70.09 ± 4.81	127.5 ± 4.47	178.7 ± 6.99	1.82	23.53 ± 0.68
	30-60 min	93.09 ± 6.39	179.4 ± 6.28	273.4 ± 10.69	1.93	10.18 ± 0.3
	1-2 h	129.5 ± 8.89	245.2 ± 8.59	380.8 ± 14.89	1.89	5.19 ± 0.15
	2-4 h	177.1 ± 12.15	330.3 ± 11.57	509.8 ± 19.94	1.87	2.86 ± 0.09
	4-8 h	181.1 ± 12.43	377.2 ± 13.21	605.3 ± 23.67	2.08	1.86 ± 0.06
	8-24 h	207.5 ± 14.24	426.5 ± 14.93	649.6 ± 25.4	2.06	0.46 ± 0.02
